# An automated haematology analyzer XN-30 distinguishes developmental stages of falciparum malaria parasite cultured in vitro

**DOI:** 10.1186/s12936-018-2208-6

**Published:** 2018-02-02

**Authors:** Takahiro Tougan, Yuhgi Suzuki, Sawako Itagaki, Munehisa Izuka, Yuji Toya, Kinya Uchihashi, Toshihiro Horii

**Affiliations:** 1 Department of Molecular Protozoology, Research Institute for Microbial Diseases, Osaka University, 3-1 Yamadaoka, Suita, Osaka 565-0871 Japan; 2 Sysmex Corporation, 4-4-4 Takatsukadai Nishiku, Kobe, Hyogo 651-2271 Japan

**Keywords:** *Plasmodium falciparum*, in vitro culture, Blood stage, Flow cytometry, XN-30 analyzer

## Abstract

**Background:**

The automated haematology analyzer XN-30 (Sysmex, Kobe, Japan) easily and rapidly detects malarial parasites in clinical blood samples using flow cytometry. The XN-30 analyzer is able to distinguish each developmental stage by measuring DNA content and cell size. Thus, it was expected to be capable of quantifying the developmental stages of cultured falciparum parasite. To achieve this requirement, a modified algorithm was tested for its validity and reliability using in vitro cultured falciparum parasite.

**Results:**

The XN-30 analyzer automatically measured each developmental stage as well as total parasitaemia. Comparison of the parasitaemia obtained using the XN-30 analyzer equipped with the modified algorithm with that obtained using microscopy examination of Giemsa-stained smears revealed the greater sensitivity and reproducibility of the former. The XN-30 analyzer also detected free merozoites and purified gametocytes.

**Conclusions:**

The XN-30 analyzer allows the precise recognition and enumeration of total and each developmental stages of cultured falciparum parasites, and permits the sensitive and reproducible calculation of parasitaemia. The results indicate the potential of the XN-30 analyzer for basic research on malarial biology, anti-malarial drug discovery, and evaluation of drug efficacy.

**Electronic supplementary material:**

The online version of this article (10.1186/s12936-018-2208-6) contains supplementary material, which is available to authorized users.

## Background

Clinically, the easy and rapid detection of malarial parasites is as crucial as the diagnosis and therapeutic monitoring of malaria [1]. Microscopy of Giemsa-stained thin blood smears remains the gold standard method to quantify malaria parasites [1]. However, it is time consuming, requires experienced and skilled microscopists and quality assurance trainings. In contrast, flow cytometry is an established method that permits fast, precise, and reliable detection of the parasite load in human blood samples and in vitro cultures in the routine laboratory setting [2]. Various flow cytometry-based methods have been developed to identify parasites and their developmental stages [2–7].

The automated haematology analyzer XN-30 (Sysmex, Kobe, Japan) has been developed to provide an effective, accurate detection and quantification of infected red blood cells (iRBCs) in human blood samples. Using a blue semiconductor 405 nm laser beam it can detect small particles. At the same time, the sheath flow direct current is adopted for measuring RBC counts, platelet counts and haematocrit, while, sodium lauryl sulphate (SLS) haemoglobin detection method enables to measure haemoglobin [8]. Newly developed reagents allow partial lysis using CELLPACK DCL while the parasite remain in the RBC, and staining of nucleic acids is accomplished using Fluorocell M and Lysercell M. The XN-30 analyzer reports not only the iRBC count as “MI-RBC#”, but also its parasitaemia as “MI-RBC%” by calculating the ratio of the number of iRBCs and total RBCs in approximately 1 min. The minimum detection sensitivity of XN-30 analyzer in the LM mode that can count small number of iRBCs is 20 parasites/μL [9]. Additionally, the XN-30 analyzer recognizes each developmental stage of the parasite using the “M scattergram” and “M(SSC-FSC) scattergram” by analysing side fluorescent light (SFL, which corresponds to DNA content), forward scattered light (FSC, indicating size of iRBCs), and side scattered light (SSC, which refers to information about the internal cell structure and its content, e.g. presence of nuclei, granules) (Additional file 1: Figure S1a and [9]).

Due to these attributes, the XN-30 analyzer should be potentially capable of distinguishing each developmental stage of in vitro cultured *Plasmodium falciparum* although it was specifically developed for clinical samples. To pursue this goal, a modified algorithm was developed and installed in the analyzer.

The present study demonstrates that the XN-30 analyzer precisely, sensitively, and reproducibly recognizes each developmental stage of the cultured falciparum parasites and reports each parasitaemia. In addition, the XN-30 analyzer allows detection of free merozoites and purified gametocytes. These findings indicate that the XN-30 analyzer is useful for various aspects of malarial research including malarial biology and anti-malarial drug discovery.

## Methods

### Parasite culture

*Plasmodium falciparum* strains K1 and 3D7 were cultured in RPMI 1640 medium supplemented with 0.5 g/L l-glutamine, 5.96 g/L HEPES, 2 g/L NaHCO_3_, 50 mg/L hypoxanthine, 10 mg/L gentamicin, 10% heat-inactivated human serum, and RBCs at 3% haematocrit in an atmosphere of 5% CO_2_, 5% O_2_, and 90% N_2_ at 37 °C as previously described [10]. Ring-form iRBCs were collected using the sorbitol synchronization technique [11]. Sorbitol selectively disrupts iRBCs containing trophozoite or schizont stage and preferentially selects for RBCs with early ring stage parasites. Briefly, the culture contents collected by centrifugation at 840×*g* for 5 min were suspended in a fivefold volume of 5% d-sorbitol (Nacalai Tesque, Kyoto, Japan) and the cells were washed twice with RPMI 1640 medium to remove d-sorbitol.

### Purification of merozoites

Free merozoites were isolated as previously described [12]. Briefly, RBCs infected with late trophozoites and schizonts were enriched using 58.5% (v/v) Percoll (Sigma-Aldrich, St Louis, MO, USA) and late-stage iRBCs were collected using density centrifugation. The Percoll-purified iRBCs were further cultured by resuspension in complete medium without fresh RBCs, for 3 h. The culture was centrifuged at 840×*g* for 5 min. The supernatant was filtered through a 1.2 μm pore size syringe filter (Minisart; Sartorius Stedim Biotech SA, Aubagne, France) and the filtrate was collected as described [13].

### Purification of gametocytes

Gametocytogenesis was induced as previously reported [14] with modifications [15]. Briefly, the culture suspension was subjected to stress conditions (high parasitaemia, nutritional stress, RBC lysis product), and incubated with 11.6 mg/mL *N*-acetyl glucosamine (Sigma-Aldrich). Gametocytaemia was assessed by microscopy of Giemsa-stained smears (Additional file 2: Table S3). The developmental stages of gametocytogenesis were classified as reported [14].

### Sequential observation of parasite development

Ring-form iRBCs were collected using sorbitol treatment (see above) [11]; the treatment was repeated two times for tight synchronization. The synchronized culture was separated into three dishes and parasitaemia was measured for each dish every 4 h for 48 h using the XN-30 analyzer and by microscopy of Giemsa-stained smears as described below.

### Measurement of parasitaemia

The XN-30 analyzer was equipped with a modified algorithm for cultured falciparum parasites (prototype: software version 01-03, build 16) and used dedicated reagents (CELLPACK DCL, SULFOLYSER, Lysercell M, and Fluorocell M) (Sysmex). In this algorithm, the merozoites are eliminated from scattergrams (SFL-SSC) to avoid counting merozoites as MI-RBC in in vitro culture. The classification of the growth stage has been optimized to suit in vitro culture conditions. Approximately 100 µL of culture suspension was applied to a Capiject Capillary Blood Collection Tube (Terumo, Tokyo, Japan) and loaded onto the XN-30 analyzer as described in the instrument manual. The iRBC counts (total, MI-RBC#; ring-forms, RNG-RBC#; trophozoites, TRPZ-RBC#; schizont, SCHZ-RBC#; and merozoites, MEROZ#) and parasitaemias (total, MI-RBC%; ring-forms, RNG-RBC%; trophozoites, TRPZ-RBC%; and schizont, SCHZ-RBC%) were automatically reported.

For microscopy, a standard thin blood smear was fixed with 100% methanol for 10 min and stained with 10% Giemsa stain working solution, pH 7.2 (Merck KGaA, Darmstadt, Germany), for 13 min. Slides were observed at 1000× magnification using the model BX50 light microscope (Olympus, Tokyo, Japan). The parasitaemia values were obtained by counting the iRBCs in at least 10,000 RBCs.

### Imaging of iRBCs using imaging-flow cytometry ImageStreamX

Twenty microlitres of parasite suspension (K1 strain; 2.5% parasitaemia, 50% haematocrit) and 20 µL Fluorocell M were mixed with 1 mL Lysercell M. As a control, another parasite suspension was incubated with 20 µL Fluorocell M for 30 min at room temperature and then diluted with phosphate buffered saline (PBS). Samples were imaged using an ImageStreamX flow cytometer according to the instrument manual (Amnis-Millipore, Seattle, WA, USA). The data was analyzed using IDEAS Application v6.0 software (Amnis-Millipore). Diameters of iRBCs were measured using Image J software, version 1.8.0 (NIH, Bethesda, MD, USA).

### Statistical analyses

Statistical analyses, including coefficient of determination (R^2^), mean, standard deviation (SD), and coefficient of variation (CV = SD/mean × 100) were calculated using Excel software (Microsoft, Redmond, WA, USA).

## Results

### Confirmation of iRBC shrinkage

**Fig. 1:**
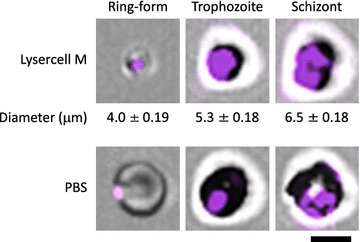
Determination of iRBC size after Lysercell M or PBS treatment using ImageStreamX. Representative images of ring-forms, trophozoites, and schizonts are shown. The iRBCs of K1 strain were treated with the Lysercell M (upper panels) or PBS (lower panels). Parasite nuclei were stained by Fluorocell M (purple). iRBC sizes are expressed as mean ± SD from three independent cells. Scale bar denotes 5 μm

**Fig. 2:**
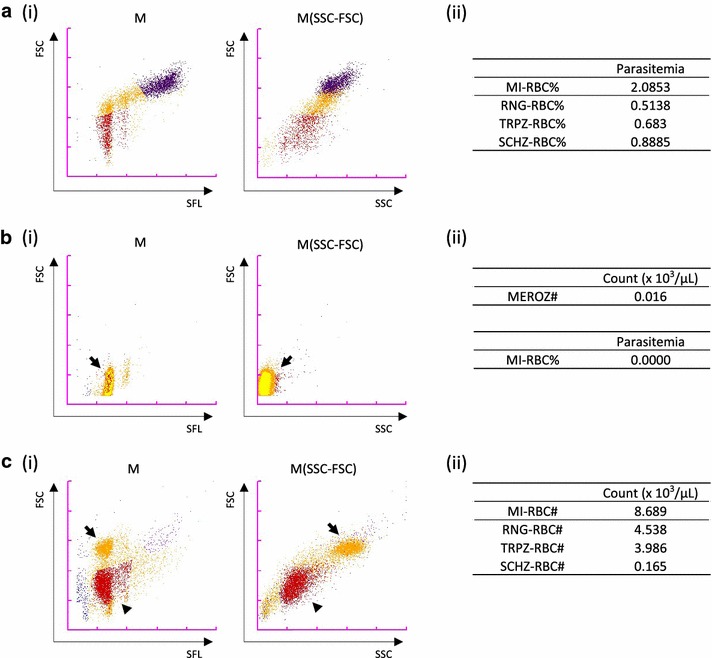
Representative data from the XN-30 analyzer. **a** Detection of ring-forms, trophozoites, and schizonts of the 3D7 strain. (i) Typical M and M(SSC-FSC) scattergrams. (ii) Parasitaemias of total and each developmental stages. Original data reported from the XN-30 analyzer are provided in Additional file 2: Table S1. **b** Detection of merozoites of K1 strain. (i) Typical M and M(SSC-FSC) scattergrams. Arrows indicate merozoites. (ii) Count of merozoites (upper panel) and parasitaemia of total iRBCs (lower panel). Original data reported from the XN-30 analyzer are provided in Additional file 2: Table S2. **c** Detection of gametocytes of 3D7 strain. (i) Typical M and M(SSC-FSC) scattergrams. Arrows and arrowheads indicate mature gametocytes and iRBCs that did not develop to gametocytes and early gametocytes, respectively. (ii) Counts of total and each developmental stages. Original data reported from the XN-30 analyzer are provided in Additional file 2: Table S4. Red, ring-forms; orange, trophozoites; purple, schizonts; yellow, merozoites; and blue, polychromatic RBCs. These experiments were performed in at least triplicate, and representative data are shown

The XN-30 analyzer recognized the developmental stages of the malarial parasites by measuring DNA content and iRBC size as indices. A previous study empirically assumed that permeabilization with Lysercell M shrunk iRBCs depending on the developmental stage of *P. falciparum* [9]. To confirm this developmental stage-dependent iRBC shrinkage, iRBCs treated with Lysercell M or PBS were imaged using the ImageStreamX flow cytometry system. Only iRBCs treated with Lysercell M shrunk in the ascending order of ring-forms, trophozoites, and schizonts (Fig. 1), indicating that the signal intensity detected by flow cytometry reflected the iRBC size depending on the developmental stage (Fig. 2a(i)).

### Detection and calculation of each developmental stage

The XN-30 analyzer recognized ring-forms, trophozoites, and schizonts on the M scattergram, as shown in Fig. 2a(i). Parasitaemias of total and each developmental stages were reported automatically (Fig. 2a(ii) and Additional file 2: Table S1). Purified merozoites were detected at the lower portion of the ring-forms on the M scattergram, and were distinguished from iRBCs on the M(SSC-FSC) scattergram (Fig. 2b and Additional file 1: Figure S2, marked by arrows; compare with Fig. 2a(i)). The XN-30 analyzer definitely detected merozoites (Fig. 2b(ii) and Additional file 2: Table S2). In case of purified gametocytes (see Additional file 2: Table S3), on the other hand, matured gametocytes (stages III/IV/V) were detected above the ring-forms and this portion was inseparable from the trophozoites on the M scattergram. This indivisibility was also found on the M(SSC-FSC) scattergram (Fig. 2c(i), marked by arrows; compare with Fig. 2a(i)). Early stage gametocytes (stages I/II) and undeveloped iRBCs were detected as ring-forms (Fig. 2c(i), marked by arrowheads). Mature and early stage gametocytes were counted as trophozoites and ring-forms, respectively (Fig. 2c(ii) and Additional file 2: Table S4; compare with Additional file 2: Table S3). The observations indicate that the XN-30 analyzer recognizes gametocytes spontaneously generated during the in vitro cultivation as ring-forms and trophozoites. Thus, although it shows inability to distinguish gametocytes from mixed cultures, the XN-30 analyzer is useful for detecting purified gametocytes.

### Comparison of parasitaemias between XN-30 analysis and microscopy of Giemsa-stained smears

**Fig. 3:**
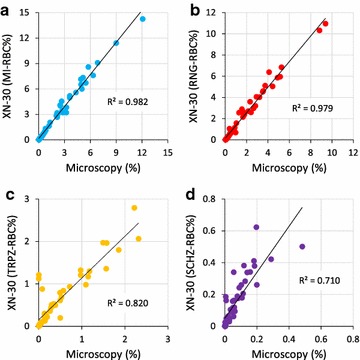
Comparison of parasitaemias for each developmental stage acquired from the XN-30 analyzer and microscopy. **a** Total iRBCs, **b** ring-form, **c** trophozoite, and **d** schizont of K1 strain. Data represents a summary of 50 biological repeats. R^2^ indicates the coefficient of determination. The diagonal line represents the regression line. All data from the XN-30 analyzer and microscopy are provided in Additional file 2: Table S5

To investigate the reliability of the XN-30 analyzer, parasitaemias of total and each developmental stages were compared between the XN-30 analysis and the gold standard microscopy examination. The correlation of total parasitaemia was high (R^2^ = 0.982; Fig. 3a), confirming prior observations (Additional file 1: Figure S1b and [9]). The coefficients of determination of parasitaemia for ring-forms, trophozoites, and schizonts were 0.979, 0.820, and 0.710, respectively (Fig. 3b–d). These results suggested that the XN-30 analyzer classifies these three developmental stages similar to the microscopic observation, although the coefficients of determination of trophozoites and schizonts were relatively low (Fig. 3c, d). This is likely due to low parasitaemia and/or morphological confusion caused by sequential change of trophozoites to schizonts.

### Sequential analysis of parasite development

**Fig. 4:**
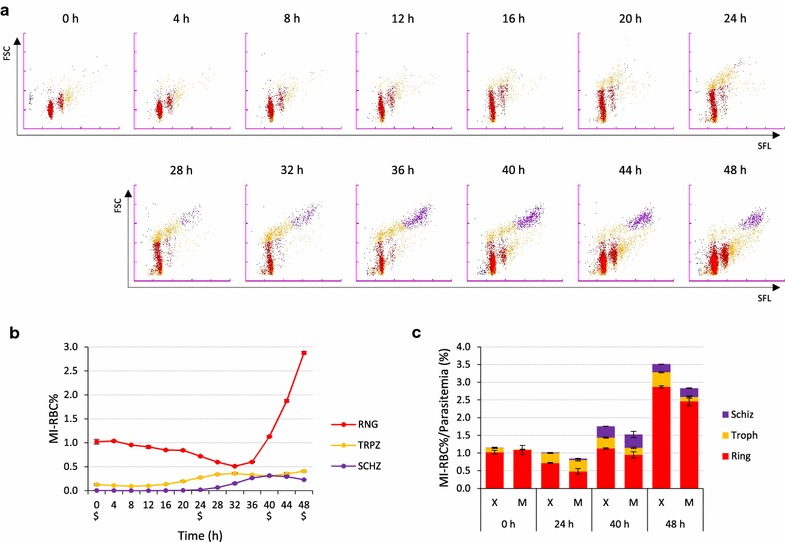
Sequential analysis of 3D7 strain after ring-form synchronization. **a** Typical M scattergrams. **b** Line graph of MI-RBC%. “$” indicates the time-point taken for comparison of parasitaemia in (**c**). Abbreviations are: RNG, ring-form; TRPZ, trophozoite; and SCHZ, schizont. **c** Bar graph of MI-RBC% and parasitaemia (%). “X” and “M” indicate the XN-30 analyzer and microscopy, respectively. Red, ring-forms; orange, trophozoites; and purple, schizonts. Results in **b** and **c** are expressed as mean ± SD from three independent dishes. All data from the XN-30 analyzer are provided in Additional file 2: Table S6

To demonstrate that the XN-30 analyzer reliably measures the proportion of trophozoite and schizont without morphological confusion, the developmental stages from tightly synchronized ring-forms was observed by both the XN-30 analyzer and microscopy. Synchronization of iRBCs to ring-forms revealed two populations on the M scattergram. The left ring-form represented one parasite and the other represented two parasites in a single RBC (Fig. 4a; 0 h). As mentioned, since the XN-30 analyzer can distinguish the amount of nucleic acid in an iRBC, cells are plotted in different populations on M scattergram [9]. Thus, the presence of single and multiple infected parasites is inferred as seen in Fig. 4a. Trophozoites were generated after 20 h and schizonts appeared after 28 h. Ring-forms of infected new RBCs increased after 36 h. The parasitaemia of ring-forms at 32 h was minimal but began to increase after 36 h as parasites infected new RBCs. The parasitaemias of trophozoites and schizonts were highest at 32 and 40 h, respectively (Fig. 4a, b). Comparison of the parasitaemia obtained with the XN-30 analyzer and Giemsa-stained microscopy revealed that the XN-30 analyzer showed smaller variability between the dishes, and slightly higher parasitaemia than microscopy (Fig. 4c and Table 1). These observations suggested that the XN-30 analyzer is more reproducible and sensitive than microscopy.

**Table 1:** Comparison of the XN-30 analysis and microscopy for the determination of parasitaemia of each stage

		Observed value of parasitaemia						XN-30 analysis/microscopy
		XN-30 analysis			Microscopy			
		Mean	SD	CV (%)	Mean	SD	CV (%)	(Total parasitaemia)
0 h	Ring	1.02	0.050	4.92	1.09	0.128	11.79	
	Troph	0.13	0.016	12.87	0.01	0.010	74.83	1.05
	Schiz	0.01	0.002	34.14	0.00	0.000	–	
24 h	Ring	0.72	0.012	1.60	0.48	0.083	17.19	
	Troph	0.28	0.007	2.57	0.32	0.027	8.36	1.20
	Schiz	0.02	0.001	6.95	0.04	0.006	16.33	
40 h	Ring	1.13	0.018	0.09	0.95	1.583	9.11	
	Troph	0.31	0.013	0.02	0.20	4.245	10.11	1.15
	Schiz	0.32	0.001	0.09	0.38	0.347	23.13	
48 h	Ring	2.88	0.029	1.01	2.46	0.130	5.28	
	Troph	0.41	0.016	3.95	0.12	0.032	27.22	1.24
	Schiz	0.23	0.002	1.03	0.25	0.013	5.27	

Data from the XN-30 analyzer are excerpted from Additional file 2: Table S6 and analyzed

*Ring* ring-form, *Troph* trophozoite, *Schiz* schizont

## Discussion

The XN-30 analyzer was developed as a specialized instrument for the detection of human malarial parasites in clinical blood samples. As complete blood count is invariably done on any febrile patient, the XN-30 analyzer is useful for the simultaneous detection of malaria parasites. Unlike the Sysmex XE and XN series which used a red 633 nm laser beam as semiconductor, the wavelength of 405 nm laser beam allows to detect smaller particles [9]. In this study, the XN-30 analyzer was tested for utilization in research settings. The analyzer separates the developmental stages of the parasite using an optimized combination of cell permeabilization and DNA staining strategies (Additional file 1: Figure S1a and [9]). Fitted by a modified algorithm, the present study provides evidence that the XN-30 analyzer precisely and reproducibly recognizes and counts each developmental stage of cultured falciparum parasites, and it reports its parasitaemia in approximately 1 min.

Brown et al. reported the detection of cultured falciparum parasite using flow cytometry in 1980 [16]. Since then, various single-, two-, and three-colour methods of flow cytometry have been developed to detect and classify the developmental stages [2–7]. The XN-30 analyzer uses a single colour to measure DNA content, the dedicated reagent Lysercell M (see Fig. 1) to evaluate iRBC size, and also estimates the amount of nucleic acids distinguishing each growth stage. This distinctive technology does not require other reagents, such as fluorescently labelled antibodies. Furthermore, the XN-30 analyzer can report results in approximately 1 min in the absence of technical experience or expertise [9]. This automated distinction of the different blood stages of the parasite in 1 min provides a powerful tool for routine laboratory use, for research on malarial biology, and for anti-malarial drug discovery.

Comparison of the parasitaemia detection results of the XN-30 analyzer and Giemsa-stained microscopy revealed relatively low R^2^ values for trophozoites (0.820) and schizonts (0.710) compared with total and ring-forms (0.982 and 0.979) (Fig. 3). This difference may reflect the low parasitaemia and/or morphological confusion of the boundary between trophozoites and schizonts for both the analyzer and microscopy. Nevertheless, the XN-30 analysis displayed higher reproducibility and sensitivity than microscopy (Fig. 4c and Table 1).

The XN-30 analyzer equipped with the default algorithm was sufficient to detect ring-forms and gametocytes in human blood samples, because trophozoites and schizonts do not appear in blood infected with the falciparum parasite (Additional file 1: Figure S1a and [9]). The algorithm for in vitro culture precisely detected trophozoites, schizonts, merozoites, as well as ring-forms (Fig. 2). Although gametocytes generated spontaneously during in vitro cultivation would be recognized as ring-forms and trophozoites, the XN-30 analyzer is useful for detecting purified gametocytes (Fig. 2c). This capability should prove to be valuable for analysis of gametocytogenesis and in discovery of drugs against gametocytes. By combining more information and further refinement, the distinction of gametocytes in mixed culture and possibly the classification of malaria species can be achieved.

It is envisioned that the XN-30 automated analyzer, which precisely recognizes each developmental stage in a reproducible manner, is very beneficial for the monitoring of stage-specific effects of drugs that are being tested. To this end, over 100 drugs were tested and usability was demonstrated (Tougan, unpublished data). Additionally, the ability to distinguish ring-forms from merozoites is a valuable tool for the examination of anti-invasion strategies including drugs and antibodies. And the capability to detect purified gametocytes is valuable for analysis of gametocytogenesis and in discovery of drugs against gametocytes.

## Conclusions

The XN-30 analyzer customized with a modified algorithm allows the precise recognition and enumeration of total and each developmental stage of cultured falciparum parasites, and permits the sensitive and reproducible calculation of parasitaemia. The results indicate the potential of the XN-30 analyzer for basic research on malarial biology, anti-malarial drug discovery, and evaluation of drug efficacy. Further improvement of the system is envisioned to be able to distinguish gametocytes in mixed culture and possibly to identify mixed malaria parasite species.

## Additional files

**Additional file 1: Figure S1.** Capability of the XN-30 analyzer equipped with the default algorithm. **Figure S2**. Representative scattergrams from the XN-30 analyzer, related to Fig. 2b.

**Additional file 2: Table S1.** Counts and parasitaemias obtained from the XN-30 analyzer, related to Fig. 2a. **Table S2.** Counts and parasitaemias obtained from the XN-30 analyzer, related to Fig. 2b. **Table S3.** Confirmation of gametocytogenesis by microscopy. **Table S4.** Counts and parasitaemias obtained from the XN-30 analyzer, related to Fig. 2c. **Table S5.** Parasitaemias obtained from the XN-30 analyzer and microscopy, related to Fig. 3. **Table S6.** Counts and parasitaemias obtained from the XN-30 analyzer, related to Fig. 4 and Table 1. **Table S7.** Counts and parasitaemias obtained from the XN-30 analyzer, related to Fig. S2.

## Abbreviations

iRBC: infected red blood cell
SLS: sodium lauryl sulphate
FSC: forward scattered light
SFL: side fluorescent light
SSC: side scattered light
PBS: phosphate buffered saline
R^2^: coefficient of determination
SD: standard deviation
CV: coefficient of variation
RIMD: Research Institute for Microbial Diseases
