# Supplementary material for: An automated haematology analyzer XN-30 distinguishes developmental stages of falciparum malaria parasite cultured in vitro
Source: Malar J. 2018 Feb 2;17:59. doi: 10.1186/s12936-018-2208-6 (PMC5796453; doi:10.1186/s12936-018-2208-6)
Supplement: Supplementary file 1 — Additional file 1: Figure S1. Capability of the XN-30 analyzer equipped with the default algorithm. Figure S2. Representative scattergrams from the XN-30 analyzer, related to Fig. 2b. [file 12936_2018_2208_MOESM1_ESM.pdf]

Fig. S1

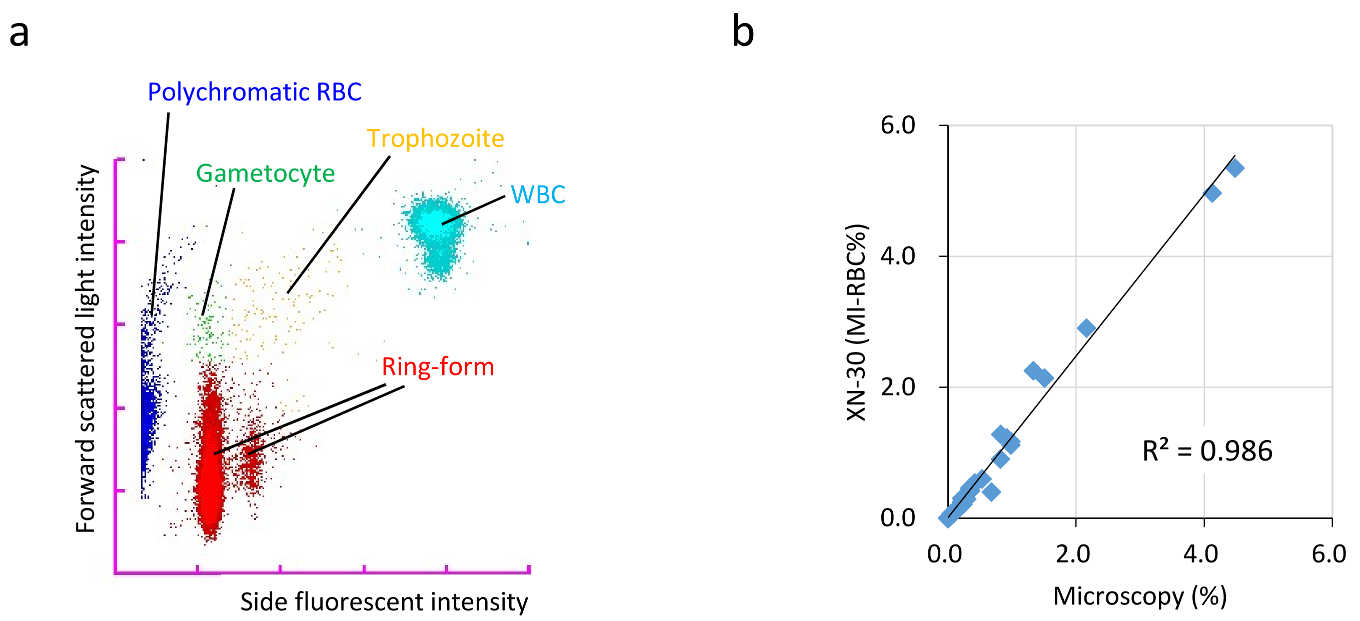

a
Polychromatic RBC
Trophozoite
Gametocyte
WBC
Ring-form
Forward scattered light intensity
Side fluorescent intensity
b
6.0
4.0
2.0
0.0
0.0
2.0
4.0
6.0
XN-30 (MI-RBC%)
Microscopy (%)
R² = 0.986

Fig. S1: Capability of the XN-30 analyzer equipped with the default algorithm. (**a**) Representative scattergram of human blood samples infected with falciparum parasites. Red, ring-forms; orange, trophozoites; green, gametocytes; blue, polychromatic RBCs; and pale blue, WBCs. (**b**) Data represents a summary of 30 biological repeats from culture suspension. R^2 indicates the coefficient of determination. The diagonal line represents the regression line. These figures are reprinted and modified with permission from the Sysmex Journal International [8].

Fig. S2

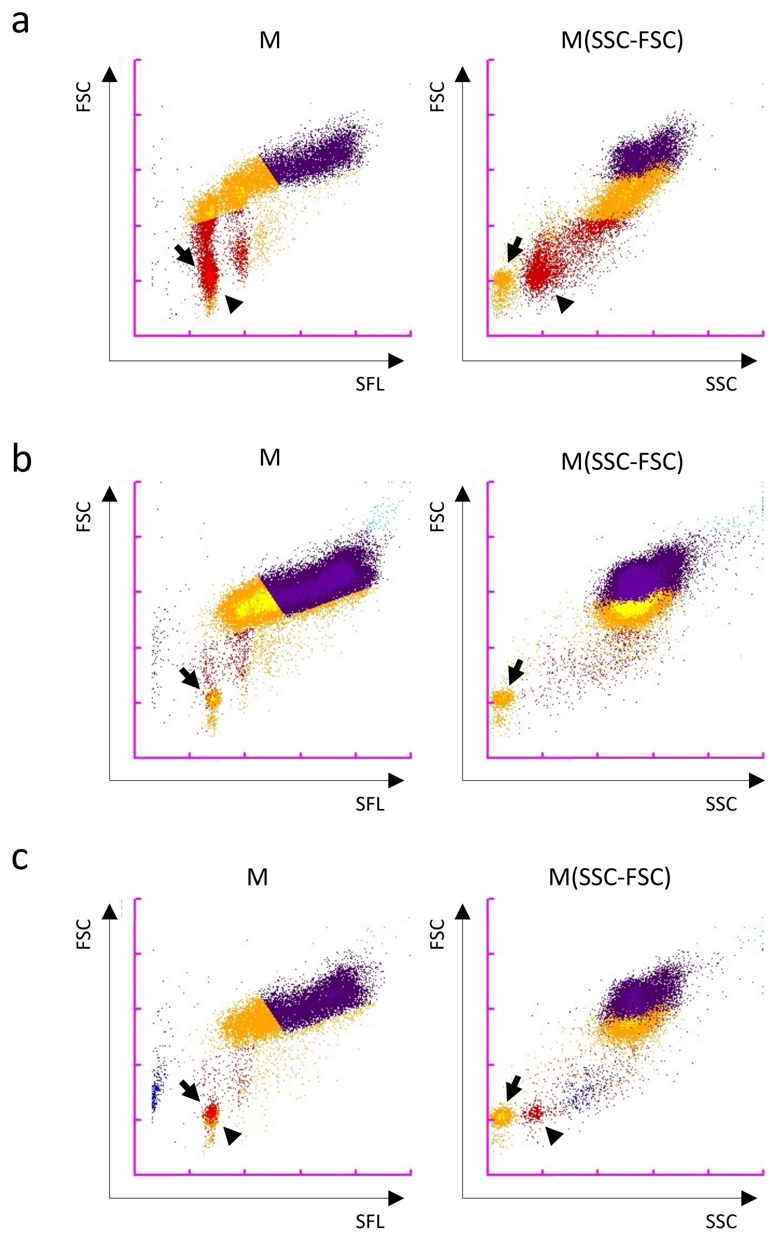
a
M
M(SSC-FSC)
FSC
SFL
SSC
b
M
M(SSC-FSC)
FSC
SFL
SSC
c
M
M(SSC-FSC)
FSC
SFL
SSC

Fig. S2: Representative scattergrams from the XN-30 analyzer, related to Fig. 2b. (**a**) Pre-Percoll suspension, (**b**) post-Percoll suspension, and (**c**) post-Percoll suspension containing RBCs. Samples were mixed and measured after 10 min. Arrows and arrowheads represent merozoites and early ring-forms, respectively. Red, ring-forms; orange, trophozoites; purple, schizonts; yellow, merozoites; and blue, polychromatic RBCs. Reported data from the XN-30 are provided in Table S7.
