# Supplementary material for: An automated haematology analyzer XN-30 distinguishes developmental stages of falciparum malaria parasite cultured in vitro
Source: Malar J. 2018 Feb 2;17:59. doi: 10.1186/s12936-018-2208-6 (PMC5796453; doi:10.1186/s12936-018-2208-6)
Supplement: Supplementary file 2 — Additional file 2: Table S1. Counts and parasitaemias obtained from the XN-30 analyzer, related to Fig. 2a. Table S2. Counts and parasitaemias obtained from the XN-30 analyzer, related to Fig. 2b. Table S3. Confirmation of gametocytogenesis by microscopy. Table S4. Counts and parasitaemias obtained from the XN-30 analyzer, related to Fig. 2c. Table S5. Parasitaemias obtained from the XN-30 analyzer and microscopy, related to Fig. 3. Table S6. Counts and parasitaemias obtained from the XN-30 analyzer, related to Fig. 4 and Table 1. Table S7. Counts and parasitaemias obtained from the XN-30 analyzer, related to Fig. S2. [file 12936_2018_2208_MOESM2_ESM.pdf]

Table S1 Counts and parasitemias obtained from the XN-30 analyzer, related to Fig. 2a

RBC($10^6/\mu L$)	MI-RBC#($10^3/\mu L$)	RNG-RBC#($10^3/\mu L$)	TRPZ-RBC#($10^3/\mu L$)	SCHZ-RBC#($10^3/\mu L$)	MEROZ#($10^3/\mu L$)
0.26	5.42	1.33	1.78	2.31	0.22

MI-RBC%	RNG-RBC%	TRPZ-RBC%	SCHZ-RBC%
2.0853	0.5138	0.683	0.8885

Table S2: Counts and parasitemias obtained from the XN-30 analyzer, related to Fig. 2b

RBC(10^6/μL)	MI-RBC#(10^3/μL)	RNG-RBC#(10^3/μL)	TRPZ-RBC#(10^3/μL)	SCHZ-RBC#(10^3/μL)	MEROZ#(10^3/μL)
0	0.064	8.463	0.978	0	0.016

MI-RBC%	RNG-RBC%	TRPZ-RBC%	SCHZ-RBC%
0	0	0	0

Table S3: Confirmation of gametocytogenesis by microscopy

Developmental stage		Parasitemia (%)*
Ring-form		0
Trophozoite		0
Schizont		0.35 ± 0.085
Gametocyte**	stage I/II (early)	0.22 ± 0.035
	stage III/IV/V (late)	3.80 ± 0.020
Undeveloped/unclassifed		3.67 ± 0.19

* Parasitemias of gametocyte were calculated based on RBC count.

** Stages were classifed according to Reference [15].

Table S4: Counts and parasitemias obtained from the XN-30 analyzer, related to Fig. 2c

RBC(10^6/μL)	MI-RBC#(10^3/μL)	RNG-RBC#(10^3/μL)	TRPZ-RBC#(10^3/μL)	SCHZ-RBC#(10^3/μL)	MEROZ#(10^3/μL)
0.1	8.689	4.538	3.986	0.165	0.793

MI-RBC%	RNG-RBC%	TRPZ-RBC%	SCHZ-RBC%
8.689	4.538	3.986	0.165

Table S5: Parasitemias obtained from the XN-30 analyzer, related to Fig. 3

	XN-30 (%)			Microscopy (%)		
Sample	RNG-RBC%	TRPZ-RBC%	SCHZ-RBC%	Ring	Trophozoite	Schizont
No.1	1.1465	0.3600	0.1197	0.8753	0.2601	0.0581
No.2	0.2369	0.0653	0.0153	0.2336	0.0515	0.0046
No.3	0.6832	0.2173	0.0643	0.5485	0.1434	0.0199
No.4	4.0675	0.8404	0.2833	2.9133	0.5709	0.1258
No.5	2.1574	0.5265	0.1624	1.6533	0.3424	0.0550
No.6	0.5480	0.1304	0.0312	0.4315	0.1162	0.0194
No.7	2.8933	1.3246	0.3388	1.5474	0.9727	0.1383
No.8	2.5715	1.1700	0.3405	1.2846	1.1461	0.0547
No.9	0.5838	0.2213	0.0254	0.4257	0.1944	0.0012
No.10	0.6477	0.2388	0.0238	0.4993	0.1659	0.0027
No.11	0.1421	0.0546	0.0075	0.1604	0.0411	0.0010
No.12	0.6003	0.2479	0.0297	0.4768	0.1762	0.0270
No.13	0.3214	0.0911	0.0079	0.2562	0.0640	0.0029
No.14	2.3900	0.4974	0.1887	1.7819	0.3851	0.0769
No.15	0.5062	0.1019	0.0448	0.4047	0.0551	0.0283
No.16	2.4891	0.5082	0.1791	1.7296	0.2974	0.1064
No.17	0.8368	0.1964	0.0909	0.6457	0.1342	0.0408
No.18	0.0510	0.0103	0.0023	0.0504	0.0137	0.0039
No.19	0.1208	0.0392	0.0044	0.1105	0.0224	0.0022
No.20	0.0230	0.0078	0.0004	0.0314	0.0016	0.0031
No.21	0.0122	0.0041	0.0006	0.0169	0.0064	0.0000
No.22	6.8571	1.9718	0.2600	5.2528	1.4922	0.1016
No.23	1.5753	0.4347	0.0500	1.0318	0.2811	0.0083
No.24	6.3930	1.9650	0.2425	4.1014	1.5759	0.0891
No.25	2.7095	0.6079	0.0947	2.1468	0.4949	0.0622
No.26	2.6892	0.9336	0.3796	1.6367	0.7182	0.1843
No.27	0.9332	0.3020	0.1052	0.6385	0.1326	0.0345
No.28	0.6963	0.1971	0.0617	0.9680	0.4993	0.0542
No.29	0.2454	0.0857	0.0325	0.1983	0.0572	0.0118
No.30	0.3243	0.0670	0.0113	0.2286	0.0335	0.0041
No.31	0.1071	0.0276	0.0067	0.0776	0.0069	0.0010
No.32	0.1826	0.0589	0.0068	0.1806	0.0201	0.0014
No.33	0.0345	0.0095	0.0023	0.0393	0.0031	0.0000
No.34	10.9600	2.7938	0.5013	9.3804	2.2192	0.4822
No.35	2.9906	0.6888	0.1582	2.6446	0.5251	0.0476
No.36	4.0338	1.2085	0.3419	3.1963	0.9727	0.1577
No.37	0.3541	0.1259	0.0482	0.3014	0.1435	0.0120
No.38	4.6094	1.2985	0.6229	3.5408	1.1756	0.1957
No.39	5.9557	1.2134	0.1857	5.1386	0.0027	0.0054
No.40	5.8722	1.1219	0.1622	4.8885	0.0000	0.0077
No.41	0.0534	0.0121	0.0021	0.0585	0.0054	0.0016
No.42	3.5950	0.9808	0.4115	2.3224	1.0931	0.1808
No.43	5.0527	0.7946	0.1762	4.4287	0.4938	0.0825
No.44	2.6056	0.5223	0.0908	2.4048	0.3682	0.0450
No.45	3.2185	0.4965	0.1169	2.8148	0.3497	0.0497
No.46	0.0578	0.0153	0.0013	0.0482	0.0127	0.0011
No.47	4.5452	1.8000	0.3817	3.4996	1.8556	0.1183
No.48	5.1167	2.0660	0.4223	3.7239	2.3156	0.2876
No.49	1.0585	1.3595	0.2610	0.3803	1.5637	0.1986
No.50	10.3304	0.8779	0.2058	8.8194	0.0808	0.1134

Table S6: Counts and parasitemias obtained from the XN-30 analyzer, related to Fig. 4 and Table 1

Sample	Counts						Parasitemisa			
	RBC($10^6/\mu L$)	MI-RBC#($10^3/\mu L$)	RNG-RBC#($10^3/\mu L$)	TRPZ-RBC#($10^3/\mu L$)	SCHZ-RBC#(103/mL)	MEROZ#($10^3/\mu L$)	MI-RBC%	RNG-RBC%	TRPZ-RBC%	SCHZ-RBC%
0 h.1	0.38	4.209	3.621	0.567	0.021	0.072	1.1075	0.9528	0.1492	0.0055
0 h.2	0.37	4.435	3.965	0.441	0.029	0.099	1.1986	1.0716	0.1192	0.0078
0 h.3	0.37	4.272	3.848	0.412	0.012	0.097	1.1547	1.0401	0.1114	0.0032
4 h.1	0.38	4.239	3.844	0.386	0.009	0.647	1.1156	1.0116	0.1016	0.0024
4 h.2	0.37	4.344	3.906	0.422	0.016	0.629	1.1741	1.0557	0.1141	0.0043
4 h.3	0.37	4.26	3.849	0.405	0.006	0.565	1.1515	1.0404	0.1095	0.0016
8 h.1	0.38	4.005	3.639	0.355	0.011	0.889	1.0539	0.9576	0.0934	0.0029
8 h.2	0.39	4.1	3.722	0.366	0.012	0.886	1.0513	0.9544	0.0938	0.0031
8 h.3	0.4	4.169	3.796	0.366	0.007	0.896	1.0421	0.9489	0.0915	0.0017
12 h.1	0.39	3.815	3.424	0.376	0.015	1.053	0.9782	0.878	0.0964	0.0038
12 h.2	0.38	3.995	3.572	0.41	0.013	1.085	1.0514	0.9401	0.1079	0.0034
12 h.3	0.39	4.007	3.589	0.416	0.002	1.077	1.0274	0.9202	0.1067	0.0005
16 h.1	0.4	3.894	3.349	0.53	0.015	1.124	0.9735	0.8372	0.1325	0.0038
16 h.2	0.4	3.998	3.467	0.516	0.015	1.134	0.9994	0.8666	0.129	0.0038
16 h.3	0.4	3.928	3.366	0.547	0.015	1.198	0.982	0.8414	0.1368	0.0038
20 h.1	0.4	4.148	3.39	0.727	0.031	1.244	1.0371	0.8475	0.1818	0.0078
20 h.2	0.4	4.138	3.281	0.835	0.022	1.201	1.0345	0.8202	0.2088	0.0055
20 h.3	0.39	4.15	3.357	0.767	0.026	1.245	1.064	0.8606	0.1967	0.0067
24 h.1	0.42	4.343	3.08	1.177	0.086	1.282	1.0341	0.7334	0.2802	0.0205
24 h.2	0.4	4.079	2.879	1.127	0.073	1.229	1.0197	0.7196	0.2818	0.0183
24 h.3	0.42	4.152	2.962	1.117	0.073	1.2	0.9886	0.7052	0.266	0.0174
28 h.1	0.42	4.182	2.523	1.379	0.28	1.095	0.9956	0.6006	0.3283	0.0667
28 h.2	0.42	4.218	2.536	1.423	0.259	1.027	1.0043	0.6038	0.3388	0.0617
28 h.3	0.42	4.263	2.469	1.504	0.29	1.038	1.015	0.5879	0.3581	0.069
32 h.1	0.43	4.211	2.177	1.446	0.588	1.044	0.9793	0.5063	0.3363	0.1367
32 h.2	0.42	4.368	2.14	1.548	0.68	1.143	1.0399	0.5094	0.3686	0.1619
32 h.3	0.42	4.385	2.146	1.577	0.662	1.063	1.0442	0.5111	0.3755	0.1576
36 h.1	0.43	5.093	2.522	1.431	1.14	1.905	1.1844	0.5865	0.3328	0.2651
36 h.2	0.43	5.199	2.633	1.403	1.163	1.923	1.2091	0.6123	0.3263	0.2705
36 h.3	0.42	5.069	2.532	1.415	1.122	1.897	1.207	0.603	0.3369	0.2671
40 h.1	0.43	7.449	4.771	1.317	1.361	5.559	1.7324	1.1096	0.3063	0.3165
40 h.2	0.43	7.72	4.96	1.391	1.369	5.8	1.7953	1.1534	0.3235	0.3184
40 h.3	0.43	7.47	4.858	1.254	1.358	5.594	1.7371	1.1297	0.2916	0.3158
44 h.1	0.43	10.757	7.908	1.607	1.242	10.35	2.5017	1.8392	0.3737	0.2888
44 h.2	0.43	11.046	8.17	1.543	1.333	11.484	2.5688	1.9	0.3588	0.31
44 h.3	0.46	11.41	8.648	1.445	1.317	11.049	2.4805	1.8801	0.3141	0.2863
48 h.1	0.43	15.042	12.193	1.845	1.004	18.798	3.4982	2.8356	0.4291	0.2335
48 h.2	0.44	15.452	12.698	1.752	1.002	18.887	3.5118	2.8859	0.3982	0.2277
48 h.3	0.44	15.517	12.777	1.727	1.013	19.046	3.5266	2.9039	0.3925	0.2302

Table S7: Counts and parasitemias obtained from the XN-30 analyzer, related to Fig. S2

	RBC($10^6/\mu L$)	MI-RBC#($10^3/\mu L$)	RNG-RBC#($10^3/\mu L$)	TRPZ-RBC#($10^3/\mu L$)	SCHZ-RBC#($10^3/\mu L$)	MEROZ#($10^3/\mu L$)
(A) Pre-Percoll	0.31	5.911	1.366	2.584	1.961	0.396
(B) Post-Percoll	0.02	15.569	0.178	4.83	10.561	0.404
(C) Post-Percoll+RBC	0.25	5.252	0.199	1.639	3.414	0.463

	MI-RBC%	RNG-RBC%	TRPZ-RBC%	SCHZ-RBC%
(A) Pre-Percoll	1.907	0.441	0.834	0.633
(B) Post-Percoll	77.848	0.893	24.150	52.805
(C) Post-Percoll+RBC	2.101	0.080	0.656	1.366
